# DDGun: an untrained method for the prediction of protein stability changes upon single and multiple point variations

**DOI:** 10.1186/s12859-019-2923-1

**Published:** 2019-07-03

**Authors:** Ludovica Montanucci, Emidio Capriotti, Yotam Frank, Nir Ben-Tal, Piero Fariselli

**Affiliations:** 1 Department of Comparative Biomedicine and Food Science (BCA), University of Padova, Viale dell’Università 16, 35020 Legnaro, Italy; 2 BioFolD Unit, Department of Pharmacy and Biotechnology (FaBiT), University of Bologna, Via Selmi 3, 40126 Bologna, Italy; 3 Department of Biochemistry and Molecular Biology, George S. Wise Faculty of Life Sciences, Tel Aviv University, Ramat Aviv, 69978 Tel Aviv, Israel; 4 Now at the Department of Medical Sciences, University of Torino, via Santena 19, 10126 Torino, Italy

**Keywords:** Unfolding free energy change, Multiple site variation, Protein stability, Protein variant

## Abstract

**Background:**

Predicting the effect of single point variations on protein stability constitutes a crucial step toward understanding the relationship between protein structure and function. To this end, several methods have been developed to predict changes in the Gibbs free energy of unfolding (∆∆G) between wild type and variant proteins, using sequence and structure information. Most of the available methods however do not exhibit the anti-symmetric prediction property, which guarantees that the predicted ∆∆G value for a variation is the exact opposite of that predicted for the reverse variation, i.e., ∆∆G(A → B) = −∆∆G(B → A), where A and B are amino acids.

**Results:**

Here we introduce simple anti-symmetric features, based on evolutionary information, which are combined to define an untrained method, DDGun (DDG untrained). DDGun is a simple approach based on evolutionary information that predicts the ∆∆G for single and multiple variations from sequence and structure information (DDGun3D). Our method achieves remarkable performance without any training on the experimental datasets, reaching Pearson correlation coefficients between predicted and measured ∆∆G values of ~ 0.5 and ~ 0.4 for single and multiple site variations, respectively. Surprisingly, DDGun performances are comparable with those of state of the art methods. DDGun also naturally predicts multiple site variations, thereby defining a benchmark method for both single site and multiple site predictors. DDGun is anti-symmetric by construction predicting the value of the ∆∆G of a reciprocal variation as almost equal (depending on the sequence profile) to -∆∆G of the direct variation. This is a valuable property that is missing in the majority of the methods.

**Conclusions:**

Evolutionary information alone combined in an untrained method can achieve remarkably high performances in the prediction of ∆∆G upon protein mutation. Non-trained approaches like DDGun represent a valid benchmark both for scoring the predictive power of the individual features and for assessing the learning capability of supervised methods.

**Electronic supplementary material:**

The online version of this article (10.1186/s12859-019-2923-1) contains supplementary material, which is available to authorized users.

## Background

The unfolding free energy difference between the wild type and mutant protein, i.e., ΔΔG = ΔG_*wildtype*_ - ΔG_*mutant*_, is the first experimental measure for studying the effect of non-synonymous variants on human health and disease, and may ultimately unravel principles of protein folding. Several methods have been developed to predict the impact of single site variants on protein stability [1], either by classifying the effect (stabilizing/destabilizing or stabilizing/destabilizing/neutral) or by quantifying the ∆∆G values (in kcal/mol).

Structure-based methods take advantage of the features representing the structural environment of the substituted residue. The combination of such features with physical and statistical potentials, improves the performance of the predictors [2]. Known structure-based algorithms include Dmutant [3], FoldX [4], I-Mutant2.0 [5, 6], CUPSAT [7], Eris [8], AUTO-MUTE [9], I-Mutant3.0 [10], PoPMuSiC [11], Pro-Maya [12], SDM [13], mCSM [14], NeEMO [15], MUpro [16] and STRUM [17]. Sequence-based methods, such as iPTREE-STAB [18], MuStab [19], INPS [20], EASE-MM [21], the sequence-based versions of I-Mutant2.0 and I-Mutant3.0 [10, 22] and PON-tstab [23], have the advantage of being applicable even when the 3D structure is not available. Although, in general, sequence-based predictors are less accurate than structure-based ones, some sequence-based methods, especially those exploiting evolutionary information, show comparable performances to structure-based tools [20].

Some structure-based predictors rely on the scores derived from different force-fields that represent the energetic contribution to protein stability. These values can be linearly combined through weights that fit the experimentally determined ∆∆G (such as in FoldX and PoPMuSiC). However, most predictors (such as MUpro, mCSM) use a combination of structural and evolutionary information features to train machine learning methods on data sets of experimentally determined ∆∆G. Machine learning approaches are also implemented in sequence-based predictors (such as in INPS). More recently, ensemble predictors (Duet [24]) and metapredictors (iStable [25]) have also been developed.

A critical assessment of the performances of all these methods is a difficult task, because they are trained on different data sets, and cross-validation procedures are often not explicitly described. However, when tested on independent data sets, not including variations used in the training step, the prediction performances of the state-of the-art tools reach Pearson correlation coefficients ranging from 0.5 to 0.7.

Most of the predictors are trained on subsets of the ProTherm database [26], which is a collection of ∆∆G values and other thermodynamic measures of protein stability. The vast majority of recorded variations in most of the currently available data sets are destabilizing; for example, in the ProTherm database more than 75% of the variations are destabilizing. Thus, predictors that do not consider the data set unbalance show a prediction bias toward destabilizing variations and a lack of anti-symmetry in the prediction of direct and reciprocal variations [27].

The physics of the thermodynamic process of folding/unfolding imposes that the ∆∆G value of changing residue *A* to residue *B* in position *p*, is the opposite (−∆∆G) of the reverse change, i.e., *B* to *A*. This defines perfect anti-symmetry. Two measures are commonly used to assess the anti-symmetrical property of ∆∆G predictors. The first measure is the Pearson correlation coefficient between the direct and the corresponding inverse variations. Given that we expect reciprocal ∆∆G values for reciprocal variations, that is ∆∆G(*A* → *B*) = −∆∆G(*B* → *A*), a perfectly anti-symmetrical predictor should produce a correlation between direct and inverse variations equal to − 1. A second measure to assess anti-symmetry is the average bias defined in the Methods section*.* The average bias estimates the deviation (in kcal/mol) from the perfect anti-symmetry in a given data set. Two recent papers compiled two balanced data sets comprising the same number of direct and reciprocal variations to test the anti-symmetrical property [28, 29]. The results showed that 15 tested methods returned biased and poor anti-symmetrical predictions. Indeed, the correlation coefficient between the direct and corresponding reverse variations ranges roughly from 0 to − 0.75, while the bias ranges from − 0.32 kcal/mol to − 0.99 kcal/mol.

While there are several predictors of ∆∆G upon single site variation, to the best of our knowledge, only Maestro [30] and FoldX [4] allow the predictions of ∆∆G upon multiple site variants. Maestro is a machine learning method while FoldX is based on a linear combination of physical and statistical potentials whose weights were chosen to fit experimental ∆∆G values. Dealing with multiple site variations adds another level of complexity beyond the prediction of the effect of a single variant on protein stability. The understanding of the complex interplay between a set of variants requires the learning of many types of combination effects (compensatory, additive, following linear or nonlinear combinations, threshold effects, etc.).

In this paper we introduce DDGun a simple method based on the combination of anti-symmetric features for predicting the ∆∆G upon variation. DDGun is an untrained method that can be seen as a benchmark for testing new anti-symmetric predictors with more complex input features. We develop two versions of the method: DDGun that relies on sequence-based features only, and DDGun3D that includes also structure-based features.

## Results

DDGun is a baseline approach that predicts ∆∆G through a linear combination of scores derived from sequence and structural features. The three following scores are based purely on sequence data:

1. the difference between the wild type and mutant residue in the Blosum62 substitution matrix (*s*_*Bl*_);
2. the difference in the interaction energy (measured through the Skolnick statistical potential [31]) between the wild-type and substituted residue with their sequence neighbours within a 2-residue window (*s*_*Sk*_);
3. the difference in the hydrophobicity between wild type and mutant residues according to the Kyte-Doolittle scale (*s*_*Hp*_).

We also developed, a structure-based version of DDGun (DDGun3D) adding two structure-based terms in the input features. The first structural term represents the difference in the interaction energy (measured through the Bastolla statistical potential [32]) between the wild-type and mutant residue with its structural neighbours (*s*_*BV*_). The second structural term is the relative solvent accessibility of the residue (*ac*), computed as the current accessibility divided by its maximum value. The first four scores are linearly combined while the latter is used to modulate the mutation effect with the residue accessibility. This effect is obtained by multiplying the total score by (1-*ac*). For a better tuning of the predictions of also fully accessible residues (*ac* = 1), the modulation factor was set to (1- *ac* + ε), where ε was arbitrarily set to 0.1. As before, we intentionally did not optimize the parameter ε to maximize performance, since we aim to develop a simple untrained baseline tool.

All first four scores described above were weighted through the profile built on the multiple sequence alignment of the protein and its homologues. Instead of taking the mere differences of the Blosum62/hydrophobic/energetic terms between the wild type and the mutant residues, we sum the differences of these terms over all possible amino acids, weighted by their frequencies, in the given position in the multiple sequence alignment of the query and homologues. Equations 1, 2, and 3 (see Methods) show that, given the same profile, the scores are anti-symmetrical by construction.

To define the weights of the linear combination of the scores, we investigated the correlations between the scores and the ∆∆G values in different data sets. The correlation between these scores and the ∆∆G of single point variations are reported in Table 1.

**Table 1:** Pearson correlation between scores and ∆∆G

Data set	VariBench 1564 variants	Broom et al. 605 variants	S2648 2648 variants	P53 42 variants	Myoglobin 134 variants
Score					
*s*_*Bl*_	0.269	0.354	0.284	0.636	0.148
*s*_*Sk*_	0.398	0.423	0.387	0.328	0.454
*s*_*Hp*_	0.248	0.263	0.298	0.143	0.500
*s*_*BV*_	0.452	0.581	0.497	0.423	0.548

The Pearson correlation coefficient and the scores were calculated as described in the Method section. The composition of the data sets is summarized in the *Data sets* section

For the implementation of our methods the weights were chosen to be proportional to the correlation between each score and the ∆∆G values in the high quality VariBench data set [23]. The procedure for the calculation of the weights is summarized in the Methods section (Eq. 5).

The sequence-based version of DDGun, takes as input only *s*_*Bl*_, *s*_*Sk*_ and *s*_*Hp*_ computed from the protein sequence alone (after normalization, Eq. 6), while DDGun3D uses a linear combination of all four scores whose weights were derived as described above (Eq. 7). The ∆∆G prediction returned by DDGun3D was then obtained by multiplying the linear combination of the scores by 1.1-*ac,* where *ac* is the relative solvent accessibility of the wild type residue. This modulation has been introduced to take into consideration that solvent exposed residues tend to have a lower effect on the ∆∆G [6, 10]. Relative accessibility is only computed for the structure with the wild type residue (and no difference in its value between the wild type and mutated residue is computed). This accessibility is used as a modulator of the combined score. It scales the combined score in the direction of producing larger scores (predicting higher ∆∆G values) for variations in amino acid position with small accessibility, i.e. in buried positions, while producing lower scores (thus predicting smaller ∆∆G values) for variants in high solvent accessible positions. In summary, two structural features, the Bastolla [32] statistical potential and the solvent accessibility, were introduced in DDGun3D.

The results showed that the correlations of the ∆∆G with the defined scores depend on the selected data set (Table 1). Consequently, the selection of different data sets results in different weights of the scores and performances of our methods. To test the robustness of our approach, we defined three versions of each method in which the weights were chosen upon the correlations between the ∆∆G and the scores in the three different data sets, VariBench, Broom et al. 2017, and S2648.

It is noteworthy that the coefficients were not chosen to fit the experimental ∆∆G and maximize the performance of the method, making DDGun a full-fledged non-trained algorithm (see Methods for details).

### Performance of DDGun on single site variations

We tested the predictive capabilities of DDGun and DDGun3D on different data sets of single site variations. The first is VariBench [23], a high quality data set, and the second comprises the 605 variations manually curated in Broom et al. [33]. The third, S2648, is the largest and most widely used, allowing comparison with other methods. Finally, we added two independent data sets of variations on the P53 [24] and myoglobin [34] proteins. The 134 variations of the myoglobin protein are not included in any of the other data sets. Only 5 of the 44 variants of the P53 data set were already present in the S2648 (but not in the remaining data sets).

The performances of the DDGun methods on these data sets are summarized in Table 2. For each method (DDGun and DDGun3D) three versions are presented according to the data set upon which the weights are chosen. The last two lines of Table 2 report the averages of the Pearson correlation and RMSE of DDGun and DDGun3D whose weights are selected from the three different data sets.

**Table 2:** Performances of the sequence-based and structure-based baseline methods on single site variations data sets

Coefficients derived from	Method	VariBench 1564 variants	Broom et al. 605 variants	S2648 2648 variants	P53 42 variants	Myoglobin 134 variants
VariBench	DDGun	0.50, 1.71	0.52, 1.77	0.50, 1.40	0.70, 1.45	0.48, 1.20
	DDGun3D	0.54, 1.70	0.62, 1.68	0.57, 1.33	0.67, 1.54	0.57, 1.0
S2648	DDGun	0.50, 1.71	0.52 1.77	0.50 1.38	0.70 1.48	0.48 1.16
	DDGun3D	0.54, 1.71	0.62 1.68	0.57 1.33	0.67 1.57	0.58 0.98
Broom et al.	DDGun	0.48, 1.73	0.52, 1.78	0.49, 1.42	0.71, 1.41	0.45, 1.29
	DDGun3D	0.54, 1.69	0.62, 1.66	0.57, 1.32	0.68, 1.51	0.56, 1.0
Average	DDGun	0.49, 1.72	0.52, 1.78	0.50, 1.4	0.7, 1.45	0.47, 1.21
	DDGun3D	0.54, 1.70	0.62, 1.67	0.57, 1.33	0.67, 1.55	0.57, 0.99

The Pearson correlation coefficient and the root mean squared error (RMSE) in kcal/mol are defined in section Methods

When the weights are chosen upon the correlations of the scores and the ∆∆G on the Broom data set, which is the smallest data set, the performances of DDGun tend to be lower. When the weights are chosen on larger data sets (either VariBench or S2648) performances increases both for DDGun and DDGun3D. Interestingly, the performances are almost identical whether the weights were chosen on VariBench or on S2648. This shows that our methods are remarkably robust as long as the correlations are derived from a large data set. In summary, Table 2 shows that despite being non-trained, the two versions of DDGun achieved remarkable performance on all data sets of single site variants, reaching Pearson correlation coefficients above 0.45, for all the data sets. As expected, the performance improves with the introduction of structural features (DDGun3D).

It is worth noting that the low correlation between the RMSE and Pearson is due to the data set distribution which only affects the Pearson correlation coefficient, as recently shown [35].

### Anti-symmetry

Besides assessing the performance of DDGun on the available data sets, an additional test was carried out to estimate the anti-symmetric property of our approach. For an unbiased estimation of the anti-symmetry we used the Ssym data set proposed in Pucci et al. [28], in which the proportions of direct and inverse variations are balanced. The performance of the methods on the prediction of direct and the corresponding reverse variations are calculated as well as the correlation between them.

In Table 3 we evaluated the anti-symmetry of DDGun and DDGGun3D on the Ssym dataset [28], and we also reported the scores for the best performing methods for this specific task (PopMusicSym [28] and SDM [13]). As expected by construction, DDGun and DDGun3D showed a near-perfect anti-symmetrical property. We indeed find the same performances on direct and reciprocal variations, with − 0.99 correlation between them. Moreover, the value of the DDGun bias, − 0.01 kcal/mol, is the lowest among all the tested methods directly addressing anti-symmetry bias [28, 29].

**Table 3:** Anti-symmetry performances of DDGun on the Ssym data set [28]

Method	Performance		Anti-symmetry	
	Direct variants Pearson r, RMSE	Inverse variants Pearson r, RMSE	r_dir-inv_	<δ > (kcal/mol)
DDGun	0.48, 1.47	0.48, 1.50	-0.99	−0.007
DDGun3D	0.56, 1.42	0.53, 1.46	−0.99	−0.02
PopMusicSym^a^	0.48, 1.58	0.48, 1.62	−0.77	0.03
SDM^a^	0.51, 1.74	0.32, 2.28	−0.75	−0.32
Maestro^a^	0.52, 1.36	0.32, 2.09	−0.34	−0.58
FoldX^a^	0.63, 1.56	0.39, 2.13	−0.38,	−0.47

The Pearson correlation coefficient (r), the root mean square error (RMSE), the correlation coefficient between observed and predicted ∆∆G values (r_**dir-inv**_**),** and the bias (<δ>) are defined in the Method section. RMSE and < δ > are expressed in kcal/mol. ^a^These values are taken from Pucci et al. [28] and are the two best performing methods in terms of anti-symmetry (PopMusicSym and SDM) and the two methods that can also predict multiple variations (Maestro and FoldX)

Although the anti-symmetrical property of DDGun is obtained by construction (Eqs. 1–3), the small deviations from perfect anti-symmetry (differences in the root mean square error, and the correlation and the bias that are not exactly − 1 and 0 kcal/mol) are due to differences in the profile of the protein and inverse protein, which in Ssym are associated with different PDB entries.

DDGun3D predictions also show remarkable anti-symmetry with an optimal correlation (*r*_*dir-inv*_ = − 0.99) between direct and corresponding reverse variants, while the bias and Pearson correlation on direct and inverse variations show small differences (<δ > =-0.02 kcal/mol). Beside the different profile, in structure-based methods other anti-symmetries can be introduced by changes in the structural neighbourhood. Indeed the variation can introduce structural changes to the wild type structure that result in a change of the number and type of residues in the neighbourhood.

It has to be stressed that correlations of Table 3 are useful to assess anti-symmetry and not as an estimation of the overall performances of these methods, given that the Ssym data set has skewed subset of experimental points that are however perfectly balanced to test anti-symmetry. In summary, the results reported in Table 3 show little or no-bias and a nearly perfect anti-symmetry of DDGun and DDGun3D on single site variants.

### Performance of DDGun on multiple site variations

As far as we know, DDGun is the first method that predicts ∆∆G changes upon multiple sites variations from sequence with a simple combination procedure considering the score associated with each substitution (Eq. 8). In Table 4 we report the performances of DDGun on a dataset of multiple site variations selected from ProTherm [26]. This dataset, named PTmul, comprises 914 multiple site variations, with a number of simultaneous variations ranging from 2 to 10. The Pearson correlation coefficient of DDGun is 0.37 on the most comprehensive dataset of multiple site variations that is available to date. The drop of the correlation from 0.50 for single site variations (in VariBench) to 0.37 for multiple site variations clearly shows that the interplay between the single variations that compose the multiple variations is complex and requires a learning process. As expected, an improvement is found when structural information is introduced. Indeed DDGun3D achieved a correlation coefficient between predicted and experimental ∆∆G of 0.39 on the PTmul data set.

**Table 4:** Performances on the 914 multiple site variation from Protherm

Method	Performance			Anti-symmetry	
	Direct and Inverse Pearson r, RMSE	Direct variants Pearson r, RMSE	Inverse variants Pearson r, RMSE	r_dir-inv_	<δ > (kcal/mol)
DDGun	0.44, 2.23	0.37, 2.23	0.37, 2.23	−1.00	0.00
DDGun3D	0.45, 2.27	0.39, 2.24	0.38, 2.25	−0.99	−0.007
Maestro	0.30, 2.59	0.55, 1.96	0.08, 3.10	−0.20	−0.92
FoldX	0.44, 3.10	0.41, 2.95	0.33, 3.24	−0.71	−0.21

The Pearson correlation coefficient (r), the root mean square error (RMSE), the correlation coefficient between observed and predicted ∆∆G values (r_**dir-inv**_**),** and the bias (<δ>) are defined in the Method section (Eqs. 9–12). RMSE and < δ > are expressed in kcal/mol

Tests including inverse variations demonstrate that DDGun returns perfect anti-symmetric predictions, showing the same performance on direct and inverse variants and unbiased results. For the sequence-based method (DDGun) the analysis was straightforward and confirmed the similarity between performance achieved on single and multiple site variations data sets.

Conversely, structural modelling is required to test the degree of preservation of the anti-symmetrical property for the structure-based method. Indeed, for multiple site variation we only have the three-dimensional structure of the wild type protein that makes the prediction possible only for the direct variants. Thus, to test its performance on inverse variations, we built models of the mutated proteins through the Modeller program [36]. Table 4 shows that DDGun3D is perfectly anti-symmetrical, with a correlation between direct and inverse variations close to − 1 and a bias near 0 kcal/mol.

Using the models of the protein with multiple site variations (Additional File 1), built using Modeller, we can compare our methods to other available tools that predict the ∆∆G for multiple site variations. To the best of our knowledge there are only two such methods, Maestro and FoldX, which are both structure-based. Both outperform DDGun3D on prediction with direct variations. However, when tested on reciprocal variations, the performances of both Maestro and FoldX drop while those of DDGun3D remain stable. It can be noted that while Maestro achieves the highest performances on direct variations, its predictive capability on inverse variations is basically random, with a correlation of 0.08 that is close to 0. Its anti-symmetricity, measured as the correlation between direct and corresponding inverse variations, is 0.20, very far from perfect (− 1). In terms of anti-symmetry, FoldX is more balanced than Maestro, however its performances on multiple site variations drop from Pearson coefficient of 0.41 on the direct variations to 0.33 on the reciprocals. The correlation between FoldX predictions for direct and inverse variations is quite high, − 0.71, even if not optimal. On multiple site variations FoldX showed a high root mean squared error (RMSE> 3 kcal/mol). Conversely DDGun3D shows near perfect anti-symmetry, with a − 0.99 Pearson correlation coefficient between direct and inverse variations.

The performances of all the available methods (including DDGun and DDGun3D) on the multiple site variation data set, are lower than those obtained for single point mutations. This may be partially due to the fact that the method error sums, generating larger noise for multiple predictions. These results confirm that untrained DDGun and DDGun3D can be considered as baseline methods for benchmarking more complex tools. Nonetheless, at the current stage, DDGun compares well with the other available methods maintaining at the same time an optimal anti-symmetry. We expect that DDGun and DDGun3D performances can be further improved through learning procedures.

**Fig. 1:**
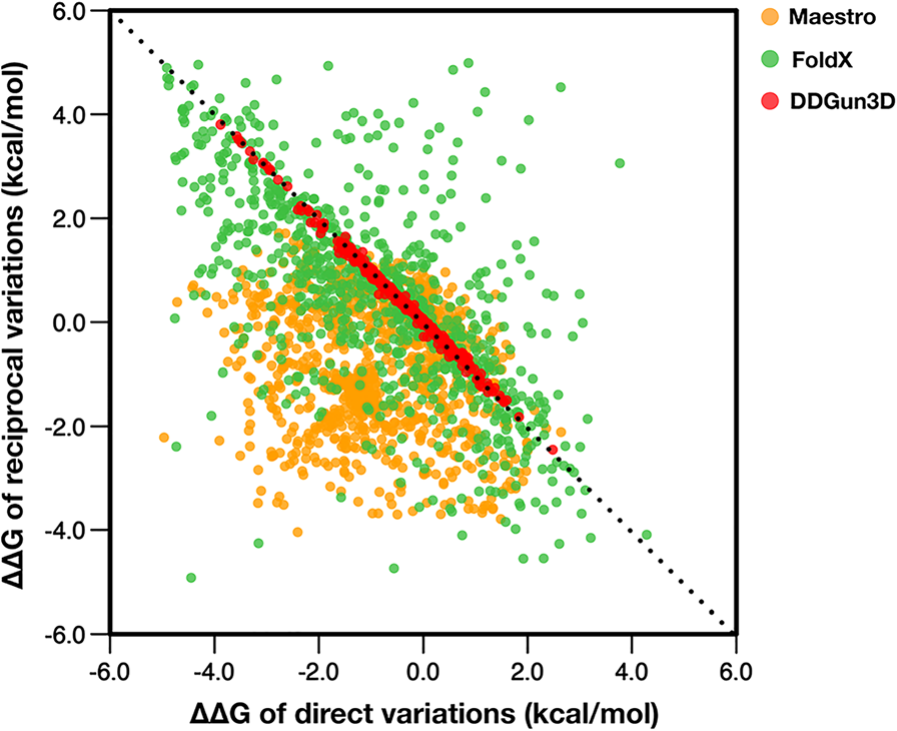
Scatter plot of the predictions of Maestro, FoldX, and DDGun3D on the PTmul data set. The x-axis shows the prediction of direct variation, and the y-axis shows the prediction for the corresponding reciprocal variation. The predictions from Maestro, FoldX and DDGun3D are plotted in yellow, green and red respectively

Figure 1 shows the predictions of direct (*x* axis) and corresponding inverse (*y* axis) ∆∆G predictions for the multiple site variations of PTmul. A perfectly anti-symmetric method would predict opposite ∆∆G values for reverse variants (∆∆G(A → B) = −∆∆G(B → A)), hence, when plotting its direct versus its reverse predictions, the points would reside exactly on the y = −x line. Deviations from that line are indicative of anti-symmetry.

The optimal anti-symmetry of DDGun3D (red) is clearly visible as its points reside very close, nearly along, the *y = −x* line, which indicates the ideal relationship of a perfectly anti-symmetrical predictor. In DDGun3D deviations from the line are very small, indicating high correlation and small bias. Conversely, predictions of Maestro (yellow) form a sparse cloud, indicating poor correlation, and its regression line is shifted by roughly 1 kcal/mol, indicating a systematic bias toward negative predictions. FoldX predictions (green) form a cloud, indicating a low anti-symmetry in terms of the correlation between direct and corresponding inverse variations. The cloud is however more evenly distributed below and above the *y = −x* axis compared to Maestro, indicating a smaller bias toward negative predictions. Indeed FoldX bias is of − 0.21 kcal/mol.

## Conclusions

The most desirable feature of a predictor is to be accurate and reliable. However, it is also very important that a predictor is compliant with the physical laws it has to simulate. In this respect, it is important for a ΔΔG predictor to be anti-symmetric with respect to the protein variations. We need predictors that can be as good as predicting protein stability changes upon variations and at the same time obtaining opposite values for the reciprocal sequence changes that bring the mutated proteins back to their respective wild types. Here we introduce simple features, based on sequence and structure information, which are anti-symmetric by construction. We show that the selected features correlate with the experimental ΔΔG measures, and also with their reverse variations -ΔΔG. We thereby combined them, defining a non-trained baseline method, which achieved remarkably high performances in the prediction of ∆∆G upon single site and multiple site variations. The results show that the evolutionary information contained in the profiles, and statistical potentials alone have high predictive power even without any training. We also provide the first method to predict ∆∆G changes upon multiple site variations from sequence information only. This will help in generalizing protein stability predictions from sequence up to genotype scale.

The high performance achieved on the single variation data set is particularly impressive in view of the recent theoretical upper bound of the prediction quality [35]. This is because even when carried out under similar pH and temperature, different measurements may sometime yield very different ∆∆G values, for example, due to changes in other experimental conditions. Machine learning approaches should suffice to improve the Pearson correlation from the ~ 0.5 value reported here without training towards the theoretical upper bound of 0.7–0.8 [35].

Finally, non-trained methods like DDGun and DDGun3D constitute a necessary benchmark to quantify the predictive capability of individual features and new prediction methods.

## Methods

### Data sets

For single point variations, the following data sets were considered: the most commonly used S2648 [11]; the high quality VariBench [23] which was integrated with the 605 manually curated variations selected in Broom et al. [33] for a total of 1900 high quality variations; a data set of variations on the P53 protein [14] and myoglobin data sets [34]. The dataset for multiple site variations was derived from ProTherm [26]. A total of 914 protein multiple site variations, with a number of simultaneous variants ranging from 2 to 10, were derived. We called this set of multiple site variations PTmul. A detailed description of the data set used in this work is reported in Table 5.

**Table 5:** Composition data sets used in this study

Data Set	Reference	Total variants	Number of proteins	Stabilizing (ΔΔG ≥ 0)	Destabilizing (ΔΔG < 0)
VariBench	Yang et al. [23]	1564	99	436	1128
Broom	Broom et al. [33]	605	58	147	458
S2648	Dehouck et al. [11]	2648	132	602	2046
P53	Pires et al. [14]	42	1	11	31
Myoglobin	Kepp et al. [34]	134	1	38	96
Ssym	Pucci et al. [28]	684	15 wild-type	342	342
PTmul	From ProTherm	914	90	310	604

**Fig. 2:**
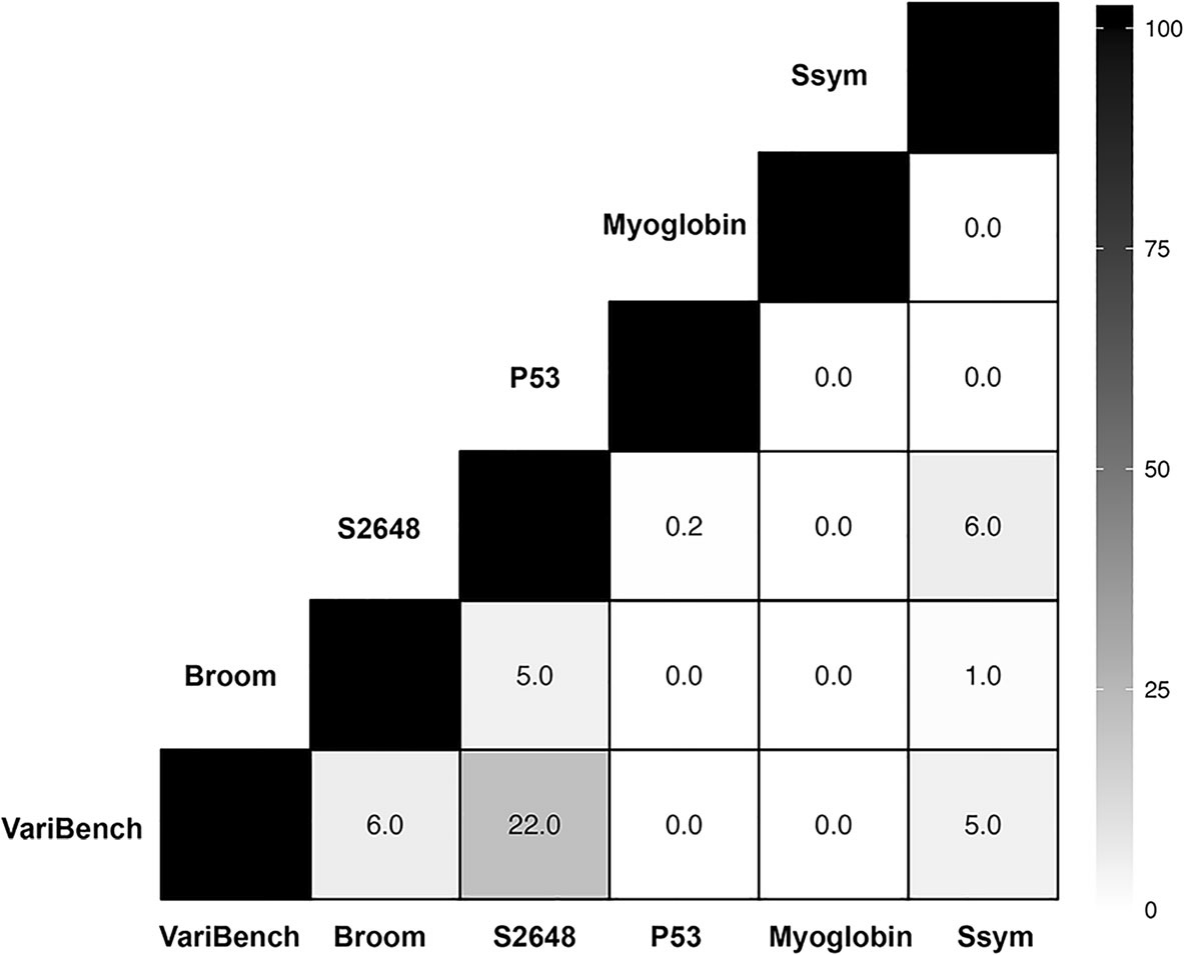
Analysis of the overlap between the single-site variant data sets. Each cell reports the percentage of the common mutations between the two corresponding data sets

In Fig. 2 we reported the overlap between the data bases considered in this work.

### Multiple sequence alignments

For each protein, identified through its PDB ID, for which we had variation data, we derived the sequence from the ATOM field of the PDB coordinate file. We built the multiple sequence alignments against the Uniprot database, release 2016 through the *hhblits* program [37] with default parameters.

### Computation of the evolutionary scores for single site variations

For each single site variation the following sequence-based scores were computed. The first score is the Blosum evolutionary score (*s*_*Bl*_) which uses the Blosum62 [38] substitution matrix to compute the difference in the substitution scores between the wild type and mutated residues. This difference is weighted through the profile, built from the multiple sequence alignments according to the following equation:

1 $$ {s}_{Bl}=\sum \limits_{i=1}^{20} prof\left({a}_i\right)\;\left(B\left({a}_i,m\right)-B\left({a}_i,w\right)\right) $$

where *w* is the wild-type residue, *m* is the variant, and *a*_*i*_ runs over the 20 standard amino acids; *B (a*_*i*_*,a*_*j*_*)* is the substitution score given by the Blosum62 matrix between the *i*-th and *j*-th amino acid; *prof (a*_*i*_*)* is the occurrence of amino acid *a*_*i*_ in the multiple sequence alignment in the considered position.

The second sequence-based score (*s*_*Sk*_) is a statistical potential developed by Skolnick et al. [31]. This score is given by the difference of the pairwise statistical potential computed between the wild type and mutated residues and their nearest neighbours in the sequence (within a two-residue window on each side). As before, instead of taking the mere difference, this score is weighted over the sequence profile as follows:

2 $$ {s}_{Sk}=\sum \limits_{j=-2,j\ne 0}^{j=2}\sum \limits_{i=1}^{20} prof\left({a}_{ij}\right)\left({P}_{Sk}\left(w,{a}_i\right)-{P}_{Sk}\left(m,{a}_i\right)\right) $$

where *prof(a*_*ij*_*)* is the profile of amino acid *a*_*i*_ in position *j* and *P*_*Sk*_*(a*_*i*_*,a*_*j*_*)* is the Skolnick potential between residues *i* and *j*.

The third hydrophobicity score (*s*_*Hp*_) measures the difference in hydrophobicity between the wild-type and mutated residues as measured by the Kyte-Doolittle hydrophobicity scale [39]. The score is weighted through the profile as follows:

3 $$ {s}_{Hp}= prof(m)K(m)- prof(w)K(w) $$

where *K(a)* is the hydrophobicity of amino acid *a* as measured by the Kyte-Doolittle scale.

The fourth structure-based score (*s*_*St*_) accounts for the structural environment of the variation, as captured by the pairwise statistical potential by Bastolla-Vendruscolo [32]. This score computes the difference in this pairwise statistical potential between the wild-type amino acid and its structural neighbours, defined within a sphere of radius 5 Å centred in the mutated site, vs. that of the variant. The profile is used to weight the contributions.

4 $$ {s}_{BV}=\sum \limits_{j\in l}\sum \limits_{i=1}^{20} prof\left({a}_{ij}\right)\left({P}_{BV}\left(w,{a}_i\right)-{P}_{BV}\left(m,{a}_i\right)\right) $$

where *I* is the set of amino acid residues in the structural neighbourhood of the substituted position; *prof(a*_*ij*_*)* is the profile of amino acid *a*_*i*_ in position *j*. *P*_*BV*_*(a*_*i*_*,a*_*j*_*)* is the Bastolla-Vendruscolo pairwise potential between residue *a*_*i*_ and *a*_*j*_.

For each mutated site, residue accessibility was computed by the DSSP program [40, 41].

### Linear combination of the scores towards ∆∆G prediction

The sequence and structure-based methods implement a linear combination of different features weighted according to their predictive power. The weights of the linear combination are proportional to the correlation between each score and the ∆∆G values from the VariBench data set [23]. More formally, the weight ${w}_{\overline{s}}$ for the score $\overline{s}$, is assigned as:

5 $$ {w}_{\overline{s}}=\frac{r\left(\overline{s},\Delta \Delta {G}_e\right)}{\sum \limits_ir\left({s}_i,\Delta \Delta {G}_e\right)} $$

where *r* is the Pearson correlation coefficient defined in Eq. 9, the sum of *s*_*i*_ at the denominator runs over all the scores to be linearly combined in an overall final score. We did not tune the parameters, nor did we change the weights for the different datasets.

### Sequence-based: DDGun

In order to combine the scores for each single site substitution, we chose a linear combination whose weights were chosen on the basis of the level of correlation of each score with the known ∆∆G for single site variations in the high-quality data set by Yang and colleagues [23].

6 $$ {s}_{seq}=0.30\cdot {s}_{Bl}+0.43\cdot {s}_{Sk}+0.27\cdot {s}_{Hp} $$

*Structure-based: DDGun3D.*

In baseline DDGun3d the final score is given by:

7 $$ {s}_{3 DA}=\left(0.20\cdot {s}_{Bl}+0.29\cdot {s}_{Sk}+0.18\cdot {s}_{Hp}+0.33\cdot {s}_{BV}\right)\left(1+\varepsilon - ac\right) $$

with ε = 0.1.

### ∆∆G prediction for multiple site variations

The above baseline method is easily adaptable to multiple site variations: indeed for each multiple-site variation we compute the score for each single site variation comprising it. Given a multiple site variation with multiplicity *M* (that is composed of *M* single site variations), let us name *s*_*s*_ the vector of *M* single site scores; *s*_*s*_ = (*s*_1_, *s*_2_, …*s*_*M*_). We compute the score for a multiple site variants as:

8 $$ {s}_{mult}=\max \left({s}_s\right)+\min \left({s}_s\right)- mean\left({s}_s\right) $$

The rationale behind this simple choice is the following. In case of more than two mutations, the most relevant points that may affect the total ΔΔG are the minimum and the maximum values, so that we decided to take their sum and centre them in the mean (by subtracting the average of the ΔΔG prediction). In case of two mutations, this reduces to the average of the two values.

### Measures of the performance

To measure the quality of the prediction, we compared the experimental (*e*) and the predicted (*p*) ∆∆G values calculating the Pearson correlation coefficient (r).

9 $$ r\left(x,y\right)=\frac{\sum \limits_{i=1}^N\left(x-\overline{x}\right)\left(y-\overline{y}\right)}{\sqrt{\sum \limits_{i=1}^N{\left(x-\overline{x}\right)}^2}\sqrt{\sum \limits_{i=1}^N{\left(y-\overline{y}\right)}^2}} $$

where x and y are the predicted and experimental ΔΔGs respectively ($\overline{x},\overline{y}$ are their average values), and the Root Mean Square Error (RMSE).

10 $$ RMSE=\sqrt{\frac{\sum \limits_{i=1}^N{\left(\Delta \Delta {G}_e-\Delta \Delta {G}_p\right)}^2}{N}} $$

To measure the anti-symmetric property of the methods we calculated the Pearson correlation coefficient between the predicted ∆∆G of the direct (*dir*) and inverse (*inv*) variations (*r*_*dir-inv*_).

11 $$ {r}_{dir- inv}=\frac{\sum \limits_{i=1}^N\left(\Delta \Delta {G}_p^{inv}-\overline{\Delta \Delta {G}_p^{inv}}\right)\left(\Delta \Delta {G}_p^{dir}-\overline{\Delta \Delta {G}_p^{dir}}\right)}{\sqrt{\sum \limits_{i=1}^N{\left(\Delta \Delta {G}_p^{dir}-\overline{\Delta \Delta {G}_p^{dir}}\right)}^2}\sqrt{\sum \limits_{i=1}^N{\left(\Delta \Delta {G}_p^{inv}-\overline{\Delta \Delta {G}_p^{inv}}\right)}^2}} $$

and the bias (<δ>).

12 $$ \left\langle \delta \right\rangle =\frac{\sum \limits_{i=1}^N\left(\Delta \Delta {G}_p^{dir}-\overline{\Delta \Delta {G}_p^{inv}}\right)}{2N} $$

According to Eqs. 11 and 12, a perfect anti-symmetric method would yield *r*_*dir-inv*_ value of − 1 and < δ > of 0 kcal/mol. All the predictions of DDGun and DDGun3D are reported in Additional file 2.

## Additional file

Additional file 1: Protein structure models used for the predictions of ∆∆G. http://folding.biofold.org/ddgun/models.tar.gz. (ZIP 1490 kb)

Additional file 2: Predictions of the unfolding ∆∆G through DDGun and DDGun3D methods. http://folding.biofold.org/ddgun/predictions.tar.gz. (ZIP 379 kb)

## Abbreviations

∆∆G: Change in the Gibbs free energy of unfolding
DDGun: Untrained sequence-based method for predicting the ∆∆G
DDGun3D: Untrained sequence and structure-based method for predicting the ∆∆G
PTmul: Data set of multiple site variations from Protherm
r: Pearson correlation coefficient
RMSE: Root Mean Square Error
S2648: Data set of 2648 single site variation from Protherm
Ssym: Data set for testing anti-symmetric prediction properties
δ: Bias in the prediction of the ∆∆G of direct and inverse variations
